# Anxiety symptoms as the central node: a network analysis of mental health sequelae in a post-COVID-19 national sample

**DOI:** 10.3389/fpsyg.2026.1748284

**Published:** 2026-03-03

**Authors:** Zhitao Yuan, Xianyang Wang, Mengyuan Yang, Lixin Zhao, Chaoxian Wang, Xiangyu Ren, Lei Ren, Na Ni, Shengjun Wu

**Affiliations:** 1 School of Public Health, Shaanxi University of Chinese Medicine, Xianyang, China; 2 Department of Military Psychology, Military Psychology Teaching and Research Center, Air Force University of Military Medicine, Xi'an, China; 3 Department of Psychology, Naval Medical University, Shanghai, China; 4 People's Liberation Army Unit, Baoji, China; 5 Military Mental Health Services & Research Center, Tianjin, China

**Keywords:** anxiety symptoms, demographic differences, mental health, network analysis, post-COVID, social support

## Abstract

**Background:**

The COVID-19 pandemic has precipitated a global mental health crisis, with the long-term effects of Post-COVID-19 syndrome presenting a complex interplay of physical and psychological symptoms. However, the dynamic network interrelationships between social support, perceived stress, and general mental health and how these networks vary across demographic groups remain inadequately explored, hindering the development of targeted interventions.

**Methods:**

To address this gap, we constructed an interactive network model using national survey data (*N* = 7,997). A Gaussian Graphical Model (GGM) was employed to estimate partial correlations among variables, and network comparison tests were conducted to examine structural differences across gender, income, residence, and marital status.

**Results:**

Anxiety and insomnia symptoms (GHA, from the General Health Questionnaire) exhibited the highest strength centrality in the network, indicating their position as the most statistically interconnected node. A strong negative correlation was identified between social support (SSS) and feelings of helplessness (PLC, from the Perceived Stress Scale), underscoring the potential protective role of social support. Notably, network invariance tests revealed significant structural variations across demographics with meaningful effect sizes. Women showed stronger stress-depression connections, whereas men exhibited a stronger negative link between social support and helplessness. Low-income groups demonstrated tighter anxiety-depression connectivity; distinct network topologies were observed between urban and rural residents, and marital status differentially influenced the prominence of tension-related versus helplessness-support connections.

**Conclusion:**

Mental health issues in the post-pandemic era exhibit distinct networked characteristics, with social support serving as a key buffer against stress. The substantial variation in symptom networks across demographic lines underscores the necessity of developing tailored, precision interventions for specific populations.

## 1 Introduction

The COVID-19 pandemic, as the most widespread global public health event of the 21st century, has not only posed a significant threat to physical health but has also triggered an unprecedented mental health crisis. This ongoing public health emergency, characterized by disease threats, social isolation measures, and socioeconomic turmoil, has had a profound negative impact on the mental health of populations worldwide. Post-COVID-19 condition (often clinically termed Post-COVID-19 syndrome) refers to the broad spectrum of signs, symptoms, and conditions that continue or develop beyond the acute phase of SARS-CoV-2 infection. The related term Long COVID is frequently used in patient-led and public discourse to describe the often-debilitating, persistent experience of these symptoms. In this manuscript, we use “post-COVID-19” as the overarching term to encompass the long-term health consequences following infection, which aligns with our study’s focus on the population-wide mental health sequelae in the aftermath of the pandemic. This condition is a multisystem disease whose occurrence does not depend on the severity of the acute infection; even individuals with mild or asymptomatic initial infections may experience persistent symptoms research indicates that this syndrome not only manifests as physical symptoms, but is also significantly associated with the occurrence and development of mental health issues such as depression and anxiety symptoms (Castanares-Zapatero et al., 2022; Fernandez-De-Las-Penas et al., 2024).

The various challenges caused by the COVID-19 pandemic are undergoing complex changes. In the post-pandemic era, public health concerns have broadened beyond acute infection to include the long-term impact on health, psychological and social pressures, disruption to the order of life, and economic uncertainty (Di Gessa et al., 2022). These symptoms not only affect individuals’ daily lives, but also place a burden on families and society. For some time to come, the global economy will remain severe, employment pressures will remain high, and the long-term effects of COVID-19 will continue. The negative emotions of fear, anxiety, and tension that the public experiences as a result, and the negative perceptions and behaviors that these emotions trigger, are likely to persist (Prashar, 2023). Critically, the nature of perceived stress in this context has evolved—it is now characterized by chronic uncertainty and multi-domain threats (e.g., health, economic, social), which may fundamentally shape the contemporary mental health symptom network.

Research indicates a significant burden of mental health sequelae among COVID-19 survivors, including clinically significant anxiety, depressive symptoms, sleep disorders, and post-traumatic stress disorder (PTSD) (Nalbandian et al., 2021). A recent meta-analysis study published by the European Centre for Disease Prevention and Control (ECDC) provides specific prevalence estimates, showing that the standardized rates of clinically significant anxiety symptoms, depressive symptoms, and PTSD among patients with post-COVID-19 syndrome in community-based cohorts are 17.2, 17.3, and 20.6%, respectively (Taher et al., 2025). These clinical phenomena align with long-term follow-up studies of survivors of previous SARS and MERS coronavirus infections, suggesting that coronavirus infection may have specific pathophysiological effects on the central nervous system. This accumulating evidence strongly indicates that in the post-pandemic era, mental health issues have evolved into a persistent and significant public health challenge.

Psychological symptoms in patients with post-COVID-19 syndrome are highly interrelated and form complex presentations (Liu et al., 2024). Longitudinal tracking studies indicate that the availability, sources, and perceived adequacy of social support were substantially altered during the pandemic instance, physical distancing measures reduced access to in-person support from friends, family, and community networks, while simultaneously increasing reliance on digital communication channels. This transformation in the social support ecosystem not only directly affects the prognosis of individual mental health, but also has a profound impact on the resilience of economic and social development through social and psychological buffering mechanisms (Berdida et al., 2025; Ulum et al., 2025). A cohort study in the UK showed that changes in the accessibility of social support services were associated with a dose–response relationship with increased anxiety levels in the elderly population (Giebel et al., 2021). A study in China found that good social support can effectively alleviate psychological stress among community residents and healthcare workers (Pei et al., 2024).

Existing research indicates that there is a close correlation between an individual’s perceived level of stress in life and mental health issues. Prolonged high-stress conditions can have a negative impact on mental health (Collins et al., 2023; Aljawarneh et al., 2024). In related studies, scholars have explored the association between perceived life stress and symptoms of depression and anxiety in adult populations, finding a significant positive correlation between the two, i.e., the higher the level of perceived stress, the more pronounced the symptoms of depression and anxiety. This conclusion supports the view that prolonged exposure to stressful environments may exacerbate mental health problems (Zhang et al., 2024). Another study examined the impact of perceived stress on post-traumatic stress disorder (PTSD). The results showed that individuals with high levels of perceived stress were more likely to exhibit symptoms related to PTSD after experiencing a traumatic event (Brownlow et al., 2018).

However, the phenomenology of perceived stress has been uniquely reconfigured in the post-COVID-19 context. Moving beyond transient or event-specific stressors, it is now often characterized by persistent, multifaceted worries about long-term health (e.g., “Long COVID” syndrome), economic precarity, and the enduring disruption of social rhythms and support networks (Jonsdottir et al., 2025). This confluence of threats fosters a state of chronic uncertainty and perceived uncontrollability, where anticipatory anxiety and feelings of helplessness become particularly salient.

In this context, a comprehensive and in-depth understanding of the current overall health status of the public is crucial for individual wellbeing, assessing the long-term impact of the pandemic, optimizing the allocation of public health resources, and building a resilient health support system (Al-Aly et al., 2024).

To achieve these aims, we employ network analysis as our methodological framework. This approach offers a powerful means for moving beyond linear associations to model the dynamic interplay among psychological factors. Network analysis has made significant progress in psychopathology research, providing a new perspective for understanding the internal mechanisms of complex psychological disorders. It has been applied to depression (Cai et al., 2024) to the study of anxiety (Zhang et al., 2024) and post-traumatic stress disorder (Williamson et al., 2024; Park and Kim, 2020), early applications in Western populations have been complemented by studies in East Asian contexts, which confirm both the universality and cultural nuance of symptom networks. For example, a network analysis of major depressive disorder in Korea identified core symptoms bridging depression and anxiety (Park and Kim, 2020).

Compared to traditional linear models, the core advantage of network analysis lies in its ability to construct symptom networks (Symptom Networks), thereby intuitively revealing the complex dynamic interactions among various psychological variables. A network analysis of COVID-19 recovers revealed that fatigue, cognitive dysfunction, and depressive mood constitute the core nodes of the symptom network, with these symptoms reinforcing each other through specific network pathways (Dong et al., 2025). This network perspective helps explain why some patients continue to experience mental health issues even after their physical symptoms have improved (Peng et al., 2023). The application of network analysis in COVID-19 mental health research also extends to optimizing intervention strategies. Network-based personalized intervention models can identify key symptom nodes and risk pathways within specific populations (Wu et al., 2025).

The growing literature on psychological networks in Asia underscores the importance of context. A multi-national Asian study of depressive disorders revealed shared and distinct symptom profiles across different countries, suggesting the influence of cultural and regional factors on network structures (Park et al., 2020). Furthermore, network comparisons between healthcare settings in India found that the “anxiety” pathway was more central for public healthcare attendees, while “somatic” symptoms were more prominent in private settings, highlighting how systemic and access-related factors can shape the presentation of distress (Sönmez et al., 2025). These findings collectively argue for the need to examine mental health networks within specific socioecological contexts, including healthcare systems and community environments.

While network studies in Asian contexts are emerging, key gaps persist in applying this approach to understand the post-pandemic mental health landscape in China. Existing research has made significant progress, but several key limitations remain. Studies employing network analysis to simultaneously model the interrelationships among perceived stress, social support, and general mental health in large community samples are scarce, particularly in the Chinese context. Most existing studies employ linear models to explore single-variable relationships, neglecting the complex network systems that these psychosocial factors may form and their potential feedback loop mechanisms. The moderating effects of demographic characteristics, such as urban–rural differences and socioeconomic income, on the network structure of psychological symptoms also require further analysis. Especially in China’s unique sociocultural context, these demographic variables may significantly alter the topological structure of psychological symptom networks by influencing resource access and perceptions of social support.

Therefore, this study aims to achieve two primary objectives, first, to construct and examine a network model delineating the interrelationships among perceived stress, social support, and general mental health symptoms in a large, national sample of Chinese adults during the post-COVID-19 era. Second, to investigate whether and how the structure of this psychosocial network varies across key demographic groups. Specifically, based on existing literature highlighting the differential impact of social determinants on mental health, we will compare network structures across: (1) gender (sexes), given known disparities in stress perception and symptom presentation; (2) income levels, considering the established link between economic strain and psychological distress; (3) urban–rural residence, accounting for differing social ecosystems and resource access; and (4) marital status, as social ties are a key component of our model.

In summary, this study employs network analysis to map the interrelationships among social support, perceived stress, and general mental health in a large Chinese adult sample. By examining network differences across key demographic groups, we aim to provide a contemporary structural blueprint of psychosocial factors. This model can serve as a valuable benchmark for future research and inform the development of precise, context-aware mental health strategies in the ongoing recovery period.

## 2 Methods

### 2.1 Participants

A cross-sectional survey was conducted nationwide from February to May 2025, utilizing a snowball sampling method. The questionnaire was distributed via Wenjuanxing, a professional online survey platform. All participants took part in the study on the basis of informed consent. Participants were screened based on lie detector questions in the questionnaire and underwent a rigorous quality control process. After excluding data that failed the attention screening, a total of 7,997 valid questionnaires were selected. All participants in this survey participated on the basis of informed consent, and those who completed the questionnaire were rewarded with a red envelope. A baseline survey questionnaire was used to collect information on residents’ gender, age, educational attainment, marital status, occupation, per capita annual income, place of residence, and occupation. This study was approved by the Ethics Committee of the Air Force Medical University of the People’s Liberation Army of China, approval number KY20234187-1.

### 2.2 Scale

The Chinese version of the Multidimensional Scale of Perceived Social Support (MSPSS) was employed to evaluate participants’ subjective perceptions of social support adequacy. The scale comprises 12 items distributed equally among three subscales: support from family, friends, and a significant other (e.g., partners, mentors). Participants indicated their agreement with each statement on a 7-point Likert scale ranging from 1 (very strongly disagree) to 7 (very strongly agree). The sum of all items produces a total score ranging from 12 to 84, with elevated scores denoting more robust perceived social support, consistent with prior validation studies (Koğar and Yılmaz, 2024; Calderón et al., 2021). For the current sample, the overall MSPSS exhibited exceptional internal consistency, with a Cronbach’s alpha coefficient of 0.985.

The Chinese version of the Perceived Stress Scale (CPSS) was used to assess the stress levels experienced by participants over the previous month. This 14-item self-report instrument comprises two dimensions: tension (7 items) and helplessness (7 items). Each item is rated on a 5-point Likert scale, ranging from 0 (never) to 4 (very often). After reverse-scoring the 7 positive items (e.g., items related to helplessness), the scores for all items are summed to yield a total score ranging from 0 to 56. A higher total score indicates a greater perceived stress level (She et al., 2021). In this study, the Cronbach’s alpha coefficient was 0.933.

The General Health Questionnaire (GHQ-28) was used to assess participants’ mental health levels. The questionnaire consists of 28 items, covering four dimensions: physical symptoms, anxiety/insomnia, social dysfunction, and severe depression, with seven questions in each dimension. A four-point scale from 1 to 4 was used, with higher scores indicating lower mental health levels (Werneke et al., 2000). The Cronbach’s *α* coefficient for this scale is 0.871.

### 2.3 Network analysis

This study employed a multi-method analytical approach to systematically examine the relationships between socio-psychological factors and mental health in the post-pandemic era. First, basic statistical analyses, including descriptive statistics, correlation analysis, and group comparisons, were conducted using SPSS software (version 21.0). Subsequently, network analysis was performed to model the intricate interplay among variables, utilizing the R statistical environment (version 4.2.3) (Epskamp et al., 2018).

We constructed a regularized partial correlation network to examine the interactions among social support, perceived stress, and mental health symptoms. The network was estimated using the mgm package (Mixed Graphical Models) (Epskamp and Fried, 2018), within a Gaussian Graphical Model (GGM) framework. Model selection was performed using the graphical least absolute shrinkage and selection operator (glasso) algorithm with the Extended Bayesian Information Criterion (EBIC) as the selection metric (Epskamp et al., 2018). The hyperparameter *γ* in the EBIC was set to 0.5. This value is standard in psychometric network analysis as it provides a balanced trade-off between network sensitivity and specificity, effectively controlling for false positive edges while retaining true connections (Friedman et al., 2008). The Fruchterman–Reingold algorithm was used to optimize the network layout, and blue and red colors were used to distinguish the direction of associations, with blue edges indicating positive correlations and red edges indicating negative correlations. Thicker edges correspond to stronger correlations. Nodes with strong correlations are arranged close to each other, while nodes with fewer connections are placed farther apart. Edge widths ranging from 0.1 to 0.5 pt. are used to distinguish correlation strength. These steps are implemented using the R package qgraph. The study focuses on three core metrics: strength centrality (the sum of direct connection weights of a node), intermediary centrality (the hub role of a node in network paths), and proximity centrality (the average distance between a node and other nodes) (Hofmann et al., 2016; van Borkulo et al., 2023). To ensure the reliability of the results, edge weight stability tests were conducted, 95% confidence intervals were calculated through 1,000 Bootstrap samples, centrality stability tests were conducted, network comparison analyses were conducted based on permutation tests to identify intergroup differences, and parameter stability verification was conducted using the case deletion method, with a total of 2,000 samples (Epskamp et al., 2018; Borsboom et al., 2018). The permutation-based invariance test is robust to unequal group sizes, as it does not rely on assumptions of equal variances or sample homogeneity (van Borkulo et al., 2023).

## 3 Results

### 3.1 Descriptive statistics

The survey sample covered all 31 provincial-level administrative regions in China, including 4,302 urban residents (53.8%) and 3,695 rural residents (46.2%). Age distribution was categorized according to WHO standards: 6,166 participants (77.1%) were aged 18–25, 801 (10.0%) were aged 26–30, and 553 (6.9%) were aged 31–40. The gender distribution showed 6,768 males (84.6%) and 1,229 females (15.4%).

Based on the standard cutoff points of the General Health Questionnaire, mental health levels were categorized into four grades according to scores: 0–14 points indicated a normal range, 15–28 points indicated mild psychological distress, 29–42 points indicated moderate psychological distress, and 43 points or above indicated severe psychological distress. As shown in Table 1, among the 7,997 participants, 47.8% (3,823) fell within the normal range, indicating relatively good overall mental health. However, 52.2% of participants reported varying degrees of psychological distress, including37.3% (2,983) with mild distress, 10.6% (848) with moderate distress, and4.3% (344) with severe distress.

**Table 1:** Distribution of participants by GHQ-28 symptom severity level (*n* = 7,997).

Severity level	GHQ score range	*n*	%	Cumulative %
Normal	0–14	3,823	47.8	47.8
Mild distress	15–28	2,983	37.3	85.1
Moderate distress	29–42	848	10.6	95.7
Severe distress	≥43	344	4.3	100

According to demographic variable statistics and difference analysis (Table 2), in terms of gender differences, men significantly outperform women in terms of mental health status and social support, while women exhibit higher levels of perceived stress. Rural residents have better mental health than urban residents, but urban residents face greater stress. The 26–30 age group has the best mental health status, while the elderly over 60 and adolescents under 18 have higher mental health risks. Educational attainment is significantly correlated with mental health, with college graduates performing the best and those with a junior high school education or below performing the worst. However, graduate students have poorer mental health than undergraduates. In terms of occupation, service industry workers, laborers, and merchants have the most prominent mental health issues, while freelancers face the greatest stress. Analysis of marital status shows that divorced individuals have the poorest mental health.

**Table 2:** Demographic characteristics and descriptive statistics of study variables.

Variable	Classification	*N*	Overall mental health	Perceived stress	Perceived social support
Gender	Male	6,768	16.11 ± 12.80	34.24 ± 8.36	63.85 ± 17.30
	Female	1,229	25.20 ± 12.62	39.05 ± 6.93	59.34 ± 15.44
	t-value		−20.11	−19.02	8.54
	*p*-value		<0.001	<0.001	<0.001
Affected by epidemic	Yes	4,898	19.97 ± 13.73	36.17 ± 7.96	62.09 ± 16.51
	No	3,099	13.61 ± 11.23	33.09 ± 8.57	64.85 ± 17.88
	*t*-value		21.6	16.37	−7.06
	*p*-value		<0.001	<0.001	<0.001
Place of residence	City	4,302	19.35 ± 13.71	35.73 ± 8.12	62.40 ± 16.70
	Countryside	3,695	15.35 ± 12.20	34.10 ± 8.50	64.04 ± 17.53
	*t*-value		13.67	8.79	−4.28
	*p*-value		<0.001	<0.001	<0.001
Age	<18	67	21.09 ± 16.88	38.07 ± 6.59	56.01 ± 17.99
	18–25	6,167	16.19 ± 12.32	34.70 ± 8.30	63.40 ± 17.23
	26–30	801	15.70 ± 11.82	33.51 ± 8.82	65.71 ± 17.54
	31–40	553	26.13 ± 15.50	38.17 ± 7.73	61.77 ± 14.53
	41–50	211	27.77 ± 14.83	36.88 ± 7.55	60.03 ± 14.44
	51–60	178	28.92 ± 13.77	37.43 ± 7.48	55.82 ± 16.17
	>60	21	34.38 ± 15.76	39.90 ± 4.86	47.19 ± 19.73
	*F* value		110.69	26.45	15.6
	*p*-value		<0.001	<0.001	<0.001
Educational	Junior high school and below	70	34.47 ± 18.17	40.89 ± 5.36	52.04 ± 17.94
	High school/secondary school	2,576	16.19 ± 12.36	34.65 ± 8.11	63.91 ± 16.40
	University college	2035	13.88 ± 11.92	33.43 ± 8.76	65.02 ± 18.05
	Undergraduate	3,044	19.94 ± 13.57	35.87 ± 8.10	61.66 ± 16.97
	Graduate students and above	281	25.05 ± 12.18	38.01 ± 7.61	61.85 ± 15.16
	*F* value		130.49	46.39	21.17
	*p*-value		<0.001	<0.001	<0.001
Marital status	Unmarried	6,788	16.27 ± 12.42	34.65 ± 8.33	63.45 ± 17.29
	Married	1,060	24.03 ± 15.44	36.13 ± 8.23	62.00 ± 15.92
	Divorced	149	27.21 ± 12.19	41.78 ± 5.47	58.05 ± 15.57
	*F* value		210.35	65.98	10.08
	*p*-value		<0.001	<0.001	<0.001
Occupation type	Professional	415	26.50 ± 16.27	37.49 ± 7.06	61.16 ± 16.34
	Service workers	41	28.83 ± 16.22	39.76 ± 7.73	49.29 ± 18.91
	Freelancers	139	23.86 ± 9.67	40.99 ± 5.21	61.32 ± 12.05
	Workers	48	30.04 ± 19.66	40.10 ± 6.15	54.92 ± 19.02
	Employees	154	28.68 ± 15.07	39.35 ± 6.98	57.56 ± 15.97
	Institutions, etc.	3,212	14.97 ± 11.87	33.36 ± 8.54	65.42 ± 16.83
	Students	2,770	18.47 ± 12.25	35.92 ± 7.90	61.97 ± 16.65
	Merchants	39	29.54 ± 16.22	39.26 ± 5.45	51.08 ± 15.48
	Other	1,179	15.44 ± 13.73	34.50 ± 8.59	62.66 ± 18.60
	*F* value		81.59	46.33	19.3
	*p*-value		<0.001	<0.001	<0.001
Annual per capita household income	10,000 and below	1,673	17.14 ± 13.63	35.54 ± 9.17	61.12 ± 18.65
	10–50 thousand (including 50 thousand)	3,214	16.62 ± 12.26	34.79 ± 8.40	63.42 ± 16.77
	50,000–100,000 (including 100,000)	1844	17.41 ± 13.03	34.63 ± 8.38	64.12 ± 16.48
	More than 100,000	1,266	20.36 ± 14.64	35.22 ± 8.30	63.79 ± 16.50
	*F* value		25.26	4.55	10.73
	*p*-value		<0.001	<0.003	<0.001

This severity categorization is presented for descriptive purposes to characterize the sample. The primary analyses of this study focus on comparing network structures across the pre-specified socio-demographic groups (gender, income, residence, marital status).

### 3.2 Network results

**Figure 1:**
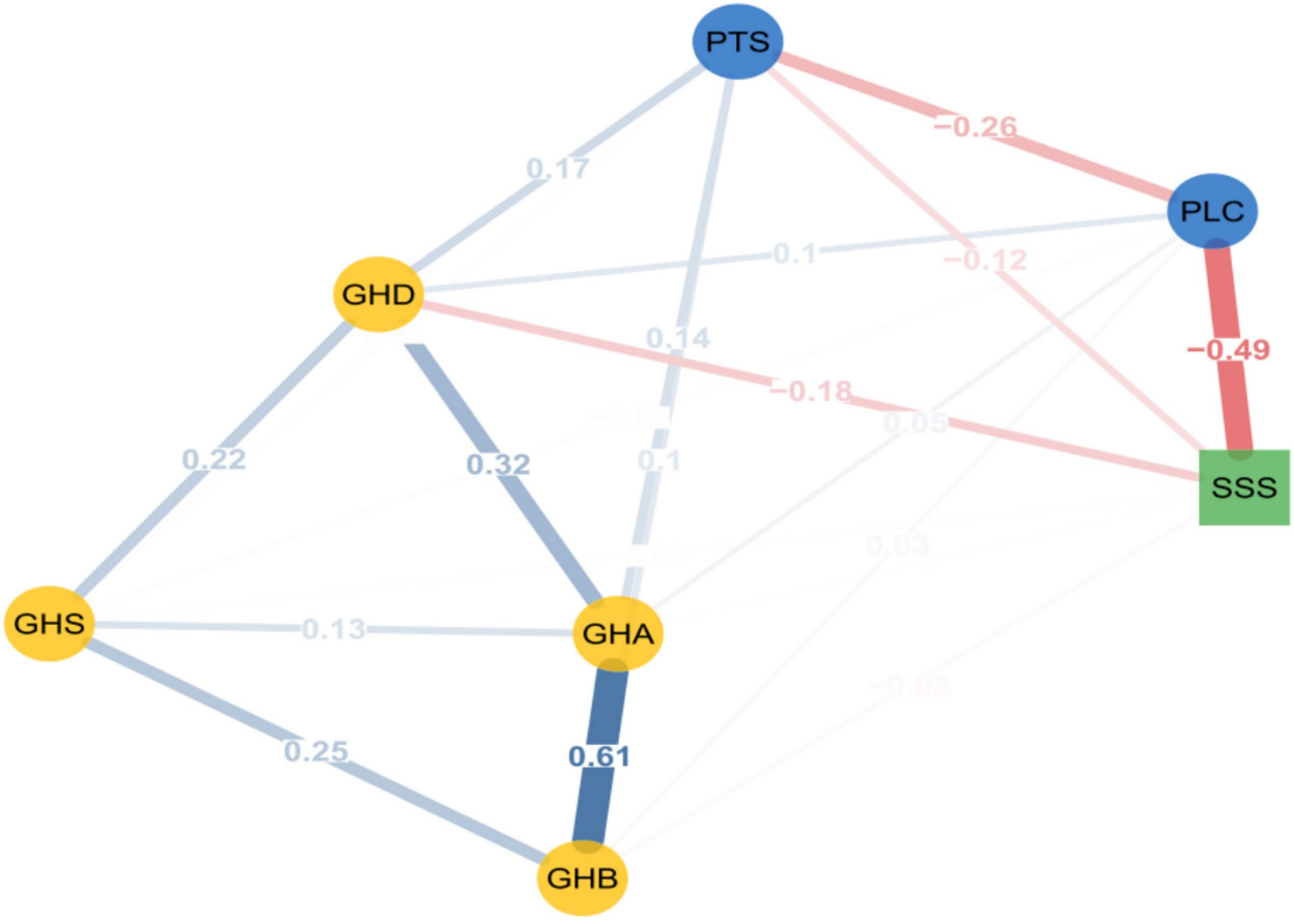
General health, social support, and perceived stress network. GHD = GHQ-28 depression symptoms; GHS = GHQ-28 social dysfunction; GHA = GHQ-28 anxiety; GHB = GHQ-28 physical symptoms; SSS = social support total score; PSS, Perceived Stress Scale; PLC = PSS sense of control; PTS = PSS tension. Blue solid lines indicate positive correlations, red dashed lines indicate negative correlations, and the wider the line, the stronger the correlation.

This study employed network analysis methods to model data from the GHQ-28 questionnaire, the social support questionnaire, and the stress perception questionnaire, revealing the complex interrelationships among various mental health variables. Psychological symptom network analysis revealed the interrelationship patterns among various mental health indicators, as shown in Figure 1. The network model includes seven nodes: perceived stress tension (PTS), perceived loss of control (PLC), physical symptoms (GHB), anxiety symptoms (GHA), social dysfunction (GHS), depressive symptoms (GHD), and social support total score (SSS).

**Figure 2:**
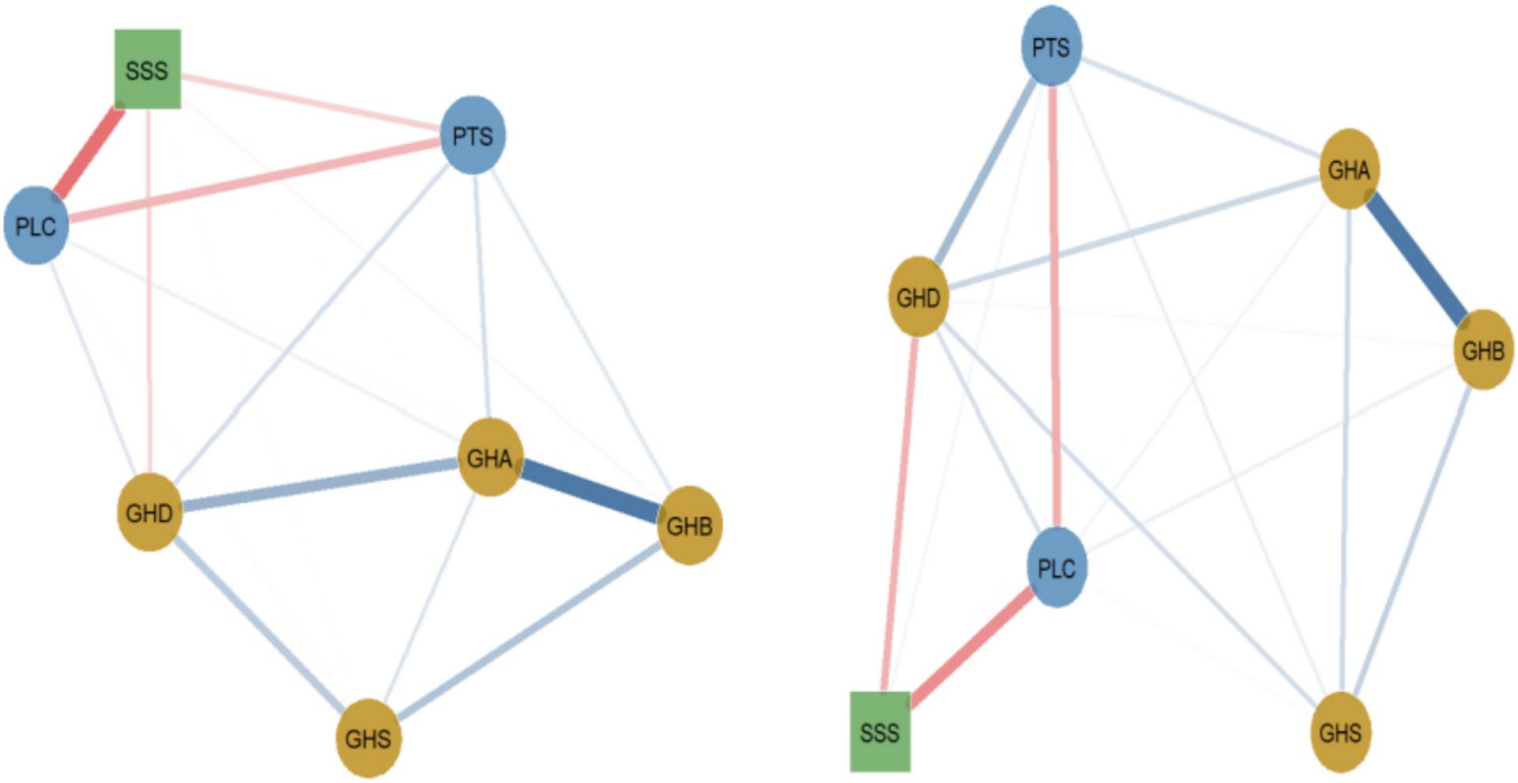
General health, social support, and perceived stress networks for men and women (left figure for men, right figure for women). Tension. Blue solid lines indicate positive correlations, red dashed lines indicate negative correlations, and the wider the line, the stronger the correlation.

The analysis results show that physical symptoms (GHB) and anxiety symptoms (GHA) exhibit the strongest connection strength (weight = 0.607), forming the most significant symptom cluster in the network, while anxiety symptoms (GHA) and depressive symptoms (GHD) form a secondary connection (weight = 0.320). Loss of control (PLC) and social support (SSS) exhibited a significant negative connection (weight = 0.492), indicating a robust inverse association between higher levels of social support and lower levels of perceived loss of control. Anxiety symptoms (GHA) exhibit the highest strength centrality in the network (weight = 1.099), positioning it as the statistically most interconnected node within the measured constructs of this model. Physical symptoms (GHB) also showed high connectivity, acting as a bridge linking somatic and psychological symptom clusters (Figure 2).

#### 3.2.1 Gender differences in network analysis

The network invariance test revealed a statistically significant difference in symptom network structure between genders, with the overall effect size in the moderate-to-large range (*M* = 0.210, *p* = 0.002). Global expected influence was 27% higher in females than in males (*S* = 0.258, *p* < 0.01), with values of 1.222 and 0.965, respectively, indicating greater overall connectivity among females.

While the core somatic-anxiety (GHB–GHA) edge was strong in both groups, its weight did not differ significantly between them (difference = 0.037, *p* > 0.05). More clinically relevant differences emerged in other pathways. The edge between perceived stress (PTS) and depression (GHD) was substantially stronger in females (weight = 0.328) than in males (weight = 0.138), with a difference of 0.190. Most notably, the protective edge between social support (SSS) and depression (GHD) was stronger in females (weight = −0.291) compared to males (weight = −0.151), with a difference of 0.140 (*p* < 0.05). Conversely, the negative edge between perceived loss of control (PLC) and social support was stronger in males (weight = −0.503) than in females (weight = −0.420), with a difference of 0.083 (*p* < 0.05).

Centrality analysis further underscored these patterns: emotional distress nodes (PTS, PLC) exhibited higher strength centrality in females (both *p* < 0.01), while social support (SSS) was more central in males (*p* < 0.01). In summary, gender differences were statistically robust and of meaningful magnitude, characterized by a female network with stronger stress-depression and support-depression links, and a male network with stronger support-coping (PLC-SSS) associations and a more central social support node.

#### 3.2.2 Income disparity network analysis

**Figure 3:**
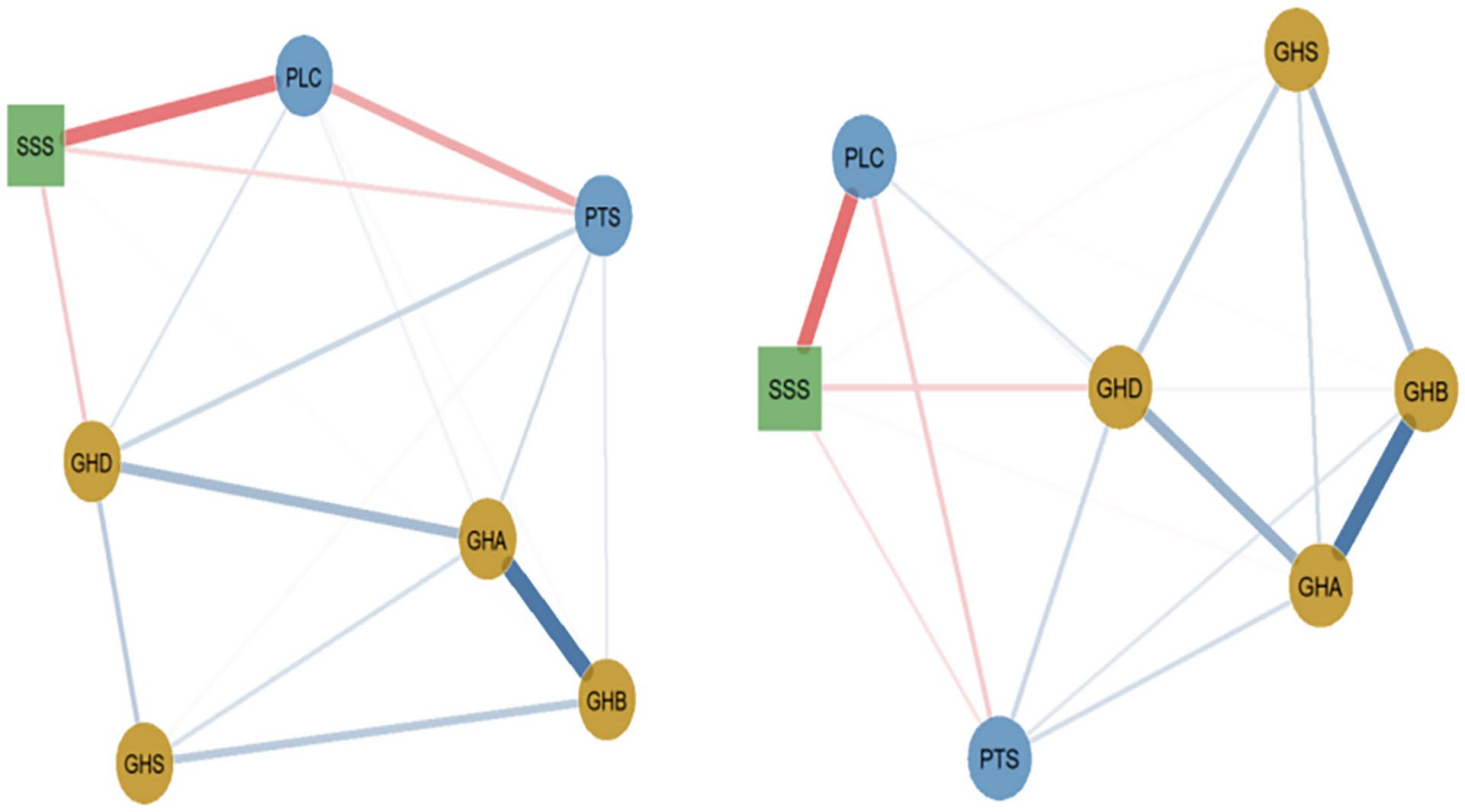
General health, social support, and perceived stress networks among low-income and high-income populations (left figure shows low-income, right figure shows high-income). Tension. Blue solid lines indicate positive correlations, red dashed lines indicate negative correlations, and the wider the line, the stronger the correlation.

A comparative analysis of the psychological symptom network characteristics of different income groups was conducted (Figure 3). Income was categorized based on the median income of urban residents in 2024 as reported by the National Bureau of Statistics. Households with an annual income exceeding 50,000 yuan were classified as high-income, while those below 50,000 yuan were classified as low-income.

The results indicated a statistically significant overall difference in network structure between income groups (*M* = 0.147, *p* = 0.002). However, the difference in global expected influence was not significant (*S* = 0.100, *p* = 0.082). Formal invariance testing identified two edges with statistically significant differences. First, the edge between perceived stress (PTS) and perceived loss of control (PLC) was stronger in the low-income group (weight = 0.307) than in the high-income group (weight = 0.160), with a significant difference (*E* = 0.147, *p* < 0.05). Second, the edge between physical symptoms (GHB) and depressive symptoms (GHD) also differed significantly between groups (*E* = 0.047, *p* < 0.05).

Other observed descriptive patterns in network weights, including those for the somatic-anxiety (GHB-GHA) and loss of control-social support (PLC-SSS) edges, were not statistically supported by invariance tests (all *p* > 0.05). Similarly, no significant differences in node centrality were found between income groups.

#### 3.2.3 Residential location differences network analysis

**Figure 4:**
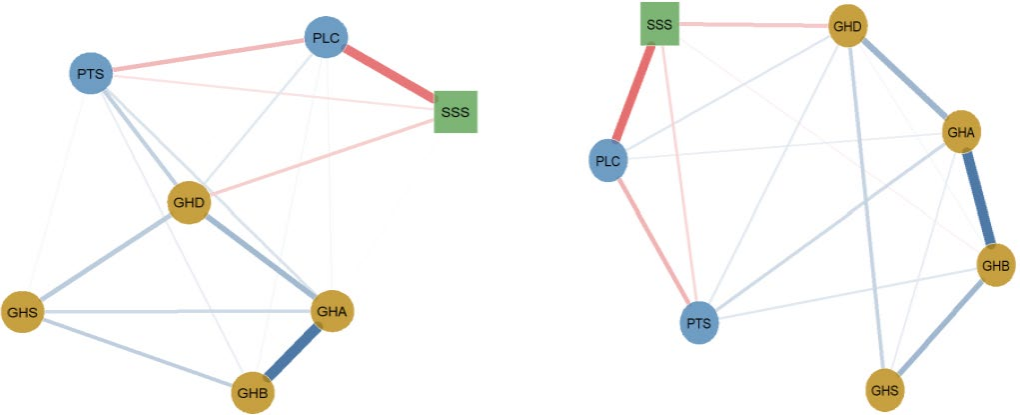
General health, social support, and perceived stress networks among urban and high-rural populations (left figure shows urban areas, right figure shows rural areas). Blue solid lines indicate positive correlations, red dashed lines indicate negative correlations, and the wider the line, the stronger the correlation.

Network analysis methods were used to compare the network characteristics of mental health symptoms between urban and rural populations. The analysis results showed (Figure 4) that the network invariance test for residential location groups reached a significant level (*M* = 0.116, *p* = 0.002), indicating a detectable but modest overall structural difference between urban and rural networks. Global expected influence was significantly higher in one group (1.119) than the other (0.891), representing a substantial 25.6% difference (*S* = 0.228, *p* = 0.002).

Both urban and rural populations exhibited the strongest connections between somatic symptoms (GHB) and anxiety symptoms (GHA) (urban: weight = 0.601; rural: weight = 0.574), as well as the second-strongest connection between anxiety symptoms and depressive symptoms (GHD) (urban: weight = 0.312; rural: weight = 0.312). The similarity in these core edges underscores a common anxiety-centered structure across residence types. Formal invariance testing, however, did not find these specific edges (GHB-GHA, GHA-GHD) to differ significantly between groups.

Further analysis revealed several edges with statistically significant differences. The edge between perceived stress (PTS) and depressive symptoms (GHD) was stronger in the urban group (weight = 0.209) than in the rural group (weight = 0.094), with a significant difference (*E* = 0.116, *p* < 0.01). Conversely, the edge between somatic symptoms (GHB) and social dysfunction (GHS) was stronger in the rural group (weight = 0.305) than in the urban group (weight = 0.167) (*E* = 0.087, p < 0.01). Other significant differences included edges between anxiety and social dysfunction (GHA–GHS: *E* = 0.083, *p* < 0.01) and between physical symptoms and social support (GHB–SSS: *E* = 0.048, *p* < 0.01). In contrast, edges such as that between perceived loss of control (PLC) and social support (SSS) showed high consistency across groups (urban: weight = 0.487; rural: weight = 0.493) and did not differ significantly (*p* > 0.05).

#### 3.2.4 Network analysis of marital status differences

Network analysis methods were used to compare the psychological health symptom networks of single and married individuals. The network invariance test demonstrated a statistically significant overall structural difference between groups (*M* = 0.138, *p* = 0.002). Global expected influence was substantially higher in single individuals (1.343) than in married individuals (0.984), representing a difference (*S* = 0.359, *p* = 0.002), indicating greater overall connectivity.

**Figure 5:**
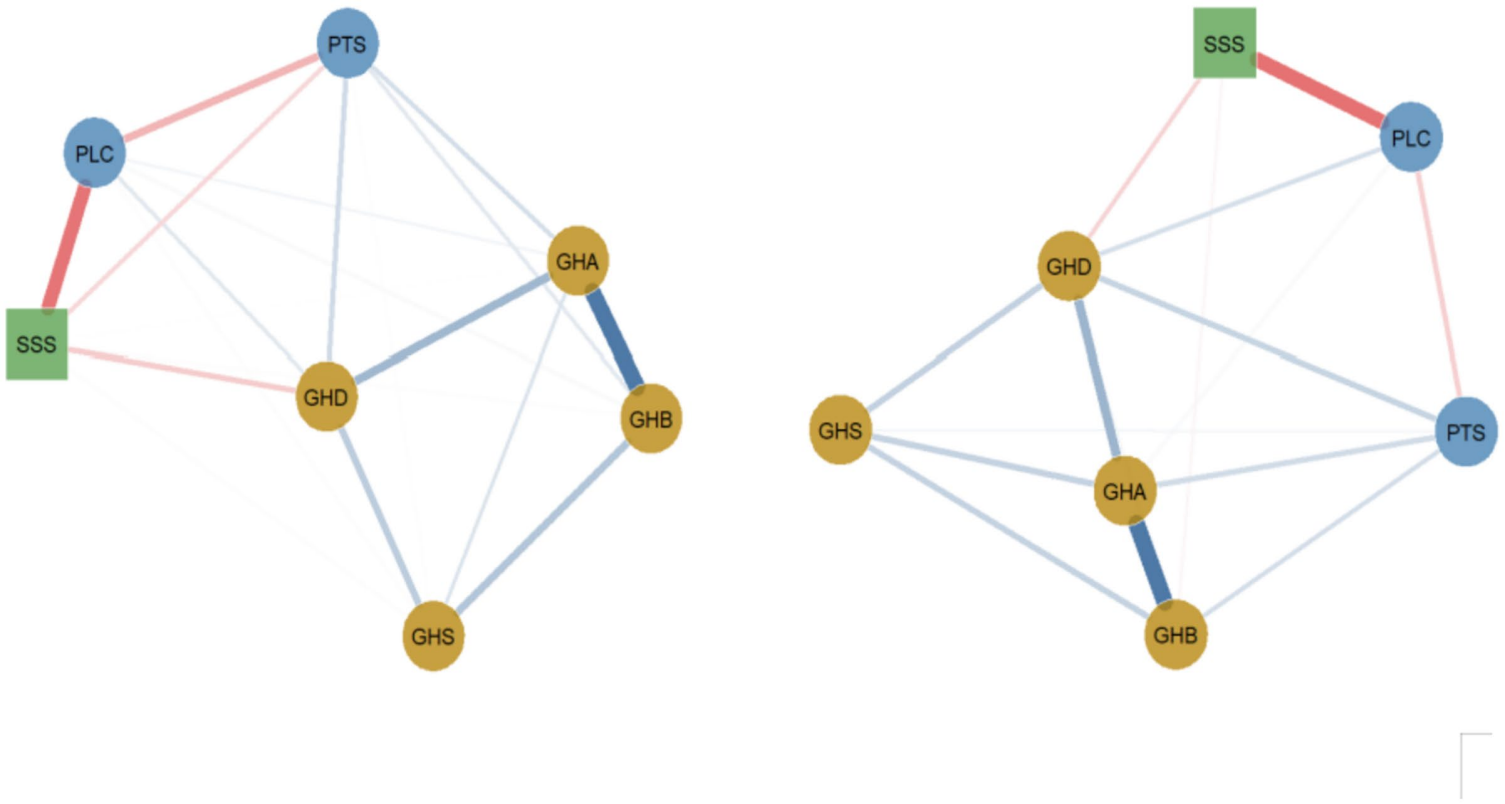
General health, social support, and perceived stress networks among single and married individuals (left figure shows single individuals, right figure shows married individuals). Tension. Blue solid lines indicate positive correlations, red dashed lines indicate negative correlations, and the wider the line, the stronger the correlation.

The estimated networks revealed descriptive patterns (Figure 5). Single individuals exhibited strong connections between somatic and anxiety symptoms (GHB–GHA: weight = 0.602) and between anxiety and depressive symptoms (GHA–GHD: weight = 0.258). Married individuals also showed robust connections in these core pathways (GHB–GHA: 0.595; GHA–GHD: 0.251). However, invariance tests indicated that these core symptom-symptom connections did not differ statistically between groups.

The only edge showing a statistically significant invariance was between perceived tension (PTS) and social support (SSS), with a stronger negative association in married individuals (*E* = 0.138, *p* < 0.05). Correspondingly, both the PTS and SSS nodes exhibited significantly different centrality between groups (both *p* < 0.01). No other edges or nodes demonstrated statistically significant differences across marital status.

## 4 Discussion

By integrating traditional statistical methods with network analysis techniques, this study identifies key intervention targets (high-strength centrality nodes), uncovers symptom transmission pathways (bridge edge analysis), and determines group-specific patterns (multi-group network comparisons). It provides new evidence on the network-mediated role of social support systems in alleviating perceived stress and maintaining mental health, as well as the influence of different demographic characteristics on network structure, thereby shedding light on the complex mechanisms underlying mental health in the post-pandemic era (van Borkulo et al., 2023). The network structure identified in our study delineates the associative landscape of mental health constructs in the post-pandemic era. In this model, the anxiety/insomnia node (GHA) displayed the highest strength centrality, indicating it was the most statistically interconnected node and showed strong partial correlations with both somatic and depressive symptoms. This pattern of association aligns with multiple studies reporting the co-occurrence of anxiety and depression symptoms among individuals with long COVID (Kleine et al., 2024; Henning et al., 2025). Our model further suggests that symptoms such as fatigue and insomnia (captured within GHA) are closely linked to a broader network of psychological and somatic distress, pointing to a potential symptom cluster that warrants further investigation. This observation is consistent with an Italian study which found that women reported higher levels of anxiety and depression and poorer quality of life across multiple psychosocial dimensions post-pandemic (Perlini et al., 2024). Importantly, sleep disturbances like insomnia have been strongly associated with elevated psychological distress in various populations (Kupferschmitt et al., 2025), which may help explain why symptoms of anxiety and insomnia (GHA) occupied such a central position in our network model.

PLC was significantly negatively correlated with social support, representing the strongest negative association observed. This pattern aligns with the well-established inverse relationship between perceived social support and perceived stress reported in the literature (Wang et al., 2025). This strong negative bridge gains specific, practical relevance in the post-COVID-19 context. When individuals face pervasive uncertainty and threats perceived as uncontrollable (e.g., long-term health risks, economic volatility), our model indicates that higher perceived social support is strongly associated with lower levels of perceived helplessness (PLC). This finding is consistent with multiple studies (Moisoglou et al., 2024) which propose that the association between social support and psychological outcomes may involve pathways related to altered stress perception or coping (Moisoglou et al., 2024).

Furthermore, the associations between social support and other variables showed gender-specific patterns in our network model, which aligns with the well-documented gender disparities in mental health we referenced in our aims. Among women, the edge between social support and depressive symptoms was stronger, indicating a tighter statistical coupling in our sample. Conversely, for men, the edge between feelings of helplessness (PLC) and social support was more pronounced. These patterns align with the existing literature on gender and mental health we referenced in our aims. The stronger link between stress and depression in women corroborates findings that women’s stress perceptions often show stronger associations with internalizing symptoms (Xiong et al., 2020). Similarly, women took on more caregiving roles during the pandemic and faced higher psychological stress (Collins et al., 2021). From the perspective of social role theory, women traditionally bear more family caregiving responsibilities. During the pandemic, this role division was further reinforced, which was closely linked to dual work-family stress for women (Zhang and Ma, 2020). Thus, our observed gender-specific network patterns may correspond to these differing psychosocial contexts. It is important to note that gender differences in network structure may be influenced by unmeasured confounding variables, and these findings should be interpreted with appropriate caution.

The symptom network model for individuals with low income was characterized by more pronounced edges linking anxiety and depressive symptoms, as well as stronger connections between feelings of tension and perceived loss of control, providing a network-level correlate to the established link between economic strain and psychological distress. This observed pattern of tighter symptom interconnectivity aligns with the broader literature demonstrating a consistent association between economic strain and elevated psychological distress. Furthermore, the stronger association observed between social dysfunction and physical symptoms in the low-income group may reflect a context in which multiple vulnerabilities—such as potential barriers to accessing healthcare—are simultaneously present. This network pattern suggests that, within this population’s reported experience, somatic health concerns and impairments in daily functioning are more closely interrelated.

Viewed in conjunction with existing evidence, the distinct, more densely interconnected symptom network observed in low-income populations in our study underscores the potential limitation of isolated, symptom-focused approaches and supports the rationale for integrated, multi-dimensional intervention strategies. The network pattern characterized by stronger anxiety-depression and social dysfunction-physical symptom links provides a systemic rationale for addressing economic and social determinants alongside psychological distress. For instance, our finding of tighter anxiety-depression connectivity aligns with experimental evidence showing that direct economic support, such as guaranteed income, can reduce psychological distress (West and Castro, 2023). Similarly, the stronger link between social dysfunction and physical symptoms highlights the need to address barriers to healthcare access, as disparities in mental health outcomes have been shown to widen during crises like the COVID-19 pandemic, particularly for lower-income families (Cho et al., 2024). Therefore, public health strategies aiming to improve mental health in this population could consider integrating economic interventions and facilitating healthcare access as foundational components of a comprehensive approach, rather than as adjuncts to conventional psychological support alone.

Network comparison tests revealed differences in the structure of psychological symptom networks between urban and rural populations, a comparison grounded in literature on differing social ecosystems. The network for urban residents was characterized by significantly stronger connections between anxiety and depressive symptoms. In contrast, the network for rural residents featured stronger connections between somatic symptoms and social dysfunction. These distinct topological patterns suggest that the organization and interrelation of mental health issues may differ across living environments. The urban pattern, with its emphasis on internalizing symptoms, may correspond to contexts with higher social-evaluative demands and complex social interactions. Conversely, the rural pattern, emphasizing the somatic-social dysfunction link, is consistent with cross-cultural observations on distress expression in close-knit, agricultural communities (West and Castro, 2023).

These network differences align with known variations in social support ecosystems. Regarding social support systems, meta-analytic evidence indicates that urban residents’ mental health levels are significantly positively correlated with the heterogeneity of their social networks, while the correlation with network size is weaker, reflecting the unique distribution pattern of urban social capital (Huang, 2022). Conversely, rural residents’ mental health primarily benefits from emotional support provided by large, homogeneous social networks, a finding that supports the differentiated expression of the social buffering hypothesis across different social environments (Stawski et al., 2008). Collectively, our network findings and this supporting literature advocate for contextually tailored public health approaches. For urban settings, interventions might prioritize accessible stress management and emotional regulation tools to address internalizing symptoms. Rural programs, however, would benefit from integrating mental health screening and education into primary care settings to de-stigmatize psychological distress, while leveraging existing strong community networks for support delivery. Future research should further explore the underlying social determinants of these urban–rural differences and their implications for mental health service policies.

Several limitations of this study warrant consideration and directly inform the interpretation of our findings. First, the cross-sectional design and the use of an online convenience sample constrain the generalizability of our results and preclude any causal inference. Although our sample captured geographic and key demographic diversity, it likely over-represents younger, digitally connected individuals and may not fully represent the broader Chinese population. Future research should validate these network structures using probability-based sampling.

The most critical limitation stems from the cross-sectional nature of our data. The estimated network reflects conditional associations at a single time point and does not elucidate temporal or causal relationships. Consequently, while anxiety symptoms (GHA) displayed the highest strength centrality in our model, this statistical property does not establish them as a mechanistic or clinical ‘core’ driver of the network. We cannot determine whether anxiety influences other symptoms, is influenced by them, or if the relationships are bidirectional. Therefore, all interpretations regarding the ‘centrality’ of anxiety should be strictly framed as hypotheses about potential dynamic importance, to be tested in longitudinal designs. The connections identified here constitute a foundational map of interrelated symptoms. We strongly advocate for future research employing longitudinal designs, such as panel networks or N-of-1 time-series studies, to investigate temporal precedence and potential causal pathways.

The scope of this study requires clear methodological positioning. Our cross-sectional design cannot establish causality or isolate specific pandemic effects from pre-existing symptom patterns. Thus, our primary aim was not causal inference but to characterize the architecture of mental health networks within the post-pandemic context—a period shaped by collective trauma and chronic stressors. This provides a contemporary snapshot of how psychosocial factors interrelate, offering a benchmark for future research and identifying potential intervention targets within the current system. Looking forward, longitudinal network studies are needed to investigate temporal dynamics, particularly whether central symptoms (e.g., anxiety) actively influence the network over time. Furthermore, network-informed interventions—such as targeting highly connected symptoms or strengthening protective links like social support—should be empirically tested in randomized trials. Fourth, our sample exhibited a significant gender imbalance (84.6% male), which reflects the demographic composition of our initial sampling frame but limits the generalizability of findings, particularly for women. Although the permutation tests used for network comparisons are robust to unequal sample sizes (van Borkulo et al., 2023), the precision of network parameter estimates (e.g., edge weights) for the smaller female subgroup is naturally lower. Future studies should endeavor to recruit more balanced samples to confirm and extend our gender-specific findings Finally, methodological advances including multi-method assessments and more diverse sampling will enhance the validity and generalizability of future network models in this field.

## 5 Conclusion

This study employed a network analysis approach to map the complex associations between social support, perceived stress, and mental health in the post-pandemic era. The findings identify anxiety symptoms as a central hub within the psychological network and illustrate a strong protective association between social support and reduced feelings of helplessness. Crucially, our analysis revealed significant heterogeneity in these network structures across demographic groups. Specifically, networks were characterized by stronger stress-depression connections in women, tighter connections between anxiety and depression symptoms in low-income groups, and distinct patterns shaped by urban–rural residence and marital status.

Collectively, these findings move beyond documenting the mere presence of symptoms and provide a novel, structural blueprint for understanding the psychological sequelae of long COVID. They underscore the necessity of moving away from one-size-fits-all interventions toward precision public health strategies. Future mental health initiatives should be tailored to specific populations, focusing on key leverage points such as mitigating anxiety symptoms and bolstering social support, while being critically informed by moderating socio-ecological contexts including socioeconomic status, living environment, and gender roles.

## Data Availability

The original contributions presented in the study are included in the article, further inquiries can be directed to the corresponding authors.
